# Astrocyte elevated gene-1 (AEG-1) is a marker for aggressive salivary gland carcinoma

**DOI:** 10.1186/1479-5876-9-205

**Published:** 2011-12-01

**Authors:** Wen-Ting Liao, Ling Guo, Yi Zhong, Yan-Heng Wu, Jun Li, Li-Bing Song

**Affiliations:** 1 State Key Laboratory of Oncology in Southern China, Guangzhou 510060, P.R. China; 2 Department of Pathology and Guangdong Provincial Key Laboratory of Molecular Tumor Pathology, Southern Medical University, Guangzhou 510515, P.R. China; 3 Department of Nasopharyngeal Cancer, Cancer Center Sun Yat-sen University, Guangzhou 510060, P.R. China; 4 Departments of Experimental Research, Cancer Center Sun Yat-sen University, Guangzhou 510060, P.R. China; 5 Department of Biochemistry, Zhongshan School of Medicine, Sun Yat-sen University, Guangzhou 510515, P.R. China; 6 First Affiliated Hospital of Jinan University, JiNan 250000, P.R. China

**Keywords:** AEG-1, Biomarker, Prognosis, Salivary gland carcinomas

## Abstract

**Background:**

Astrocyte elevated gene-1 (AEG-1) is associated with tumorigenesis and progression in diverse human cancers. The present study was aimed to investigate the clinical and prognostic significance of AEG-1 in salivary gland carcinomas (SGC).

**Methods:**

Real-time PCR and western blot analyses were employed to examine AEG-1 expression in two normal salivary gland tissues, eight SGC tissues of various clinical stages, and five pairs of primary SGC and adjacent salivary gland tissues from the same patient. Immunohistochemistry (IHC) was performed to examine AEG-1 protein expression in paraffin-embedded tissues from 141 SGC patients. Statistical analyses was applies to evaluate the diagnostic value and associations of AEG-1 expression with clinical parameters.

**Results:**

AEG-1 expression was evidently up-regulated in SGC tissues compared with that in the normal salivary gland tissues and in matched adjacent salivary gland tissues. AEG-1 protein level was positively correlated with clinical stage (*P *< 0.001), T classification (*P *= 0.008), N classification (*P *= 0.008) and M classifications (*P *= 0.006). Patients with higher AEG-1 expression had shorter overall survival time, whereas those with lower tumor AEG-1 expression had longer survival time.

**Conclusions:**

Our results suggest that AEG-1 expression is associated with SGC progression and may represent a novel and valuable predictor for prognostic evaluation of SGC patients.

## Background

Salivary gland carcinoma (SGC) is a relatively rare cancer that accounts for less than 5% of all head and neck cancers [1,2]. It is among the most complex malignancies owing to diverse histological characteristics and biological behaviors. According to the World Health Organization (WHO) classification, SGC is one of the most complex malignancies for it has up to 24 different histological subtypes [3]. Although there have been some remarkable advances, treating SGC is still challenging, and the clinical outcomes of advanced SGC have not significantly improved [4]. More than 5% of patients suffer a recurrence at the primary site and/or distant metastasis, and the incidence of occult lymph node metastasis is also high in major SGC types [5,6]. Due to the limited number of cases available in most series of salivary gland tumors, the molecular mechanism of the development and progression of SGC is still poorly understood. Therefore, it is of great value to better understand the etiology and to identify valuable diagnostic and prognostic markers as well as novel therapeutic strategies of the disease.

AEG-1 was originally discovered as a novel protein induced by HIV-1 or tumor necrosis factor-α in primary human fetal astrocytes [7-9]. In recent years, numerous researches have revealed the essential role of AEG-1 in the development and progression of cancer. Aberrant elevation of AEG-1 expression frequently occurs in human cancers, including breast cancer, glioma, melanoma, esophageal squamous cell carcinoma, prostate cancer, hepatocellular carcinoma and gastric cancer [10-16]. As a downstream target of Ha-Ras, AEG-1 has an essential role in regulating tumorigenesis, invasion, metastasis and angiogenesis [17]. AEG-1 can promote proliferation via suppression of FOXO1, induce serum-independent cell growth and suppress apoptosis through activation of PI3K-Akt signaling [18-21], and increase anchorage-independent growth of non-tumorigenic astrocytes through activation of PI3K-Akt and NF-κB pathway [18,22]. In addition, knockdown of AEG-1 inhibits the progression of prostate cancer through up-regulation of FOXO3a activity [15]. Moreover, overexpression of AEG-1 promotes tumorigenesis and progression via activation of the Wnt/β-Catenin and NF-κB pathways in hepatocellular carcinoma [11]. Further more, AEG-1 can regulate human malignant glioma invasion through up-regulation of matrix metalloproteinase-9 and activating the NF-κB signaling pathway [10,19,22,23]. These findings suggest that AEG-1 plays a dominant positive role in development and progression of diverse cancers.

However, whether AEG-1 deregulation also occurs in SGC remains unclear. To address this question, we investigated the expression of AEG-1 in SGC and evaluate its prognostic significance by correlating AEG-1 expression levels with clinicopathologic features and survival in 141 archived SGC samples.

## Methods

### Patients and tissue specimens

Paraffin-embedded, archived SGC samples were obtained from 141 patients diagnosed with SGC between January 2001 and December 2003 at the Sun Yat-sen University Cancer Center. Clinical and pathologic classification and staging were determined according to the classification criteria proposed by the WHO [3]. Clinical information for the samples is summarized in Table 1. Two normal salivary gland tissues obtained from patients with head and neck tumors undergoing surgical procedures, eight biopsies of SGC tissues and five pair of SGC tissues with matched adjacent non-cancerous salivary gland tissues were frozen and stored in liquid nitrogen until further use. For the use of these clinical materials for research purposes, prior patient consent and approval from the Institutional Research Ethics Committee were obtained.

**Table 1:** Clinicopathological characteristics of patient samples and expression of AEG-1 in salivary gland cancer

	All cases (%)		All cases (%)
**Gender**		**Histological Types**	
Male	77 (54.61)	Mucoepidermoid carcinoma	34 (24.11)
Female	64 (45.39)	Adenoid cystic carcinoma	20 (14.18)
**Age (years)**		Acinar cell carcinoma	21 (14.89)
< 48	72 (51.06)	Adenocarcinoma	23 (16.31)
≥ 48	69 (48.94)	Squamous cell carcinoma	12 (8.51)
**Clinical Stage**		Salivary duct carcinoma	16 (11.35)
I	16 (11.35)	Basal cell carcinoma	15 (10.64)
II	49 (34.75)	**Vital status (at follow-up)**	
III	34 (24.11)	Alive	95 (67.38)
IV	42 (29.79)	Death (all SGC-related)	46 (32.62)
**T classification**		**Expression of AEG-1**	
T1	17 (12.06)	Negative	5 (3.55)
T2	60 (42.55)	Positive	136 (96.45)
T3	33 (23.40)	Low expression	62 (43.97)
T4	31 (21.99)	High expression	79 (56.03)
**N classification**		**Drinking**	
N0	106 (75.18)	No	117 (82.98)
N1	17 (12.06)	Yes	24 (17.02)
N2	18 (12.77)	**Smoking**	
**M classification**		No	101 (71.63)
No	99 (70.21)	Yes	40 (28.37)
Yes	42 (29.79)		

### RNA extraction and real-time PCR

Total RNA from tissue samples were extracted using the Trizol reagent (Invitrogen, Carlsbad, CA) according to the manufacturer's instruction. Real-time PCR was performed according to standard methods as described previously [24]. Sequences of the real-time PCR primers and probes have been reported previously [13]. Expression data were normalized to the geometric mean of the housekeeping gene *GAPDH *[13] and calculated as 2^-[(Ct of *AEG-1*)-(Ct of *GAPDH*)]^, where C_t _represents the threshold cycle for each transcript.

### Western blot

Western blots were performed according to standard methods as described previously [13], using a rabbit anti-AEG-1 polyclonal antibody (1:500; Zymed). A mouse anti-*α*-Tubulin antibody (1:1,000; Sigma, Saint Louis, MI) was used as an inner control.

### Immunohistochemical (IHC) analysis

IHC analysis were carried out similarly to previously described methods [13]. Briefly, tissue sections were incubated with a rabbit anti-AEG-1 antibody (1:200; Zymed) overnight at 4°C. For negative controls, the rabbit anti-AEG-1 antibody was replaced with normal non-immune serum.

The degree of I of paraffin-embedded sections was reviewed and scored independently by two observers, based on both the proportion of positively stained tumor cells and the intensity of staining [13]. The proportion of tumor cells was scored as follows: 0 (no positive tumor cells), 1 (< 10% positive tumor cells), 2 (10-50% positive tumor cells) and 3 (> 50% positive tumor cells). The intensity of staining was graded according to the following criteria: 0 (no staining); 1 (weak staining = light yellow), 2 (moderate staining = yellow brown) and 3 (strong staining = brown). The staining index (SI) was calculated as staining intensity score × proportion of positive tumor cells. Using this method of assessment, we evaluated the expression of AEG-1 in benign salivary gland tissues and SGC lesions by determining the SI, which scores as 0, 1, 2, 3, 4, 6 and 9. Cutoff values for AEG-1 were chosen on the basis of a measure of heterogeneity with the log-rank test statistical analysis with respect to overall survival. An optimal cutoff value was identified: the SI score of ≥ 4 was used to define tumors as having high AEG-1 expression and ≤ 3 as having low expression of AEG-1.

To account for inconsistencies in IHC stain intensities, the mean optical density (MOD) method, which was used for the scoring of the staining intensity, was applied in the current study. In brief, the stained slides were evaluated at 200× magnification using the SAMBA 4000 computerized image analysis system with Immuno 4.0 quantitative program (Image Products International, Chantilly, VA). Ten representative staining fields of each tumor sample were analyzed to determine the MOD, which represented the concentration of the stain or proportion of positive pixels within the whole tissue. A negative control for each staining batch was used for background subtraction in the quantitative analysis. The data were statistically analyzed using t-test to determine the differences in average MOD values between different groups of tissues. *P *< 0.05 was considered significant.

### Statistical analyses

All statistical analyses were carried out using the SPSS 13.0 statistical software package. Comparisons between groups for statistical significance were performed with a two-tailed paired Student's t test. The chi-square test was used to analyze the relationship between AEG-1 expression and clinicopathologic features. Bivariate correlations between variables were calculated by Spearman's correlation coefficients. Survival curves were plotted by the Kaplan-Meier method and compared using the log-rank test. Survival data were evaluated using univariate and multivariate Cox regression analyses. *P *< 0.05 in all cases was considered statistically significant.

## Results

### AEG-1 is up-regulated in SGC

**Figure 1:**
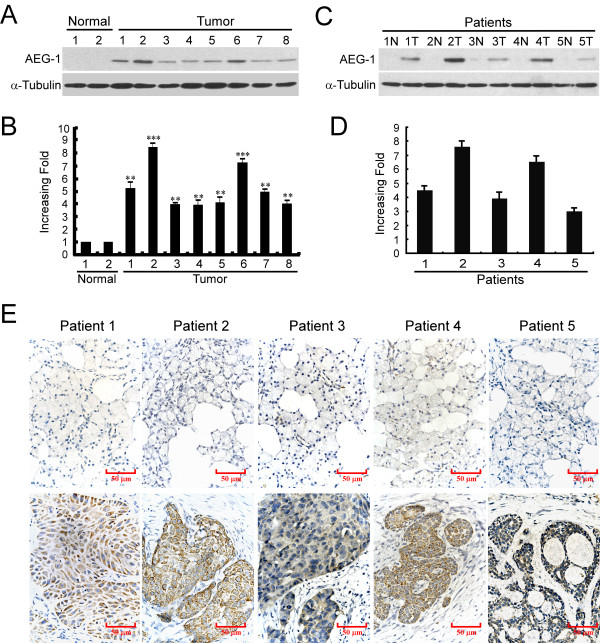
**Upregulation of AEG-1 expression in SGC tissues**. **(A and B) **Expression of AEG-1 protein and mRNA in 2 normal human salivary gland tissues and 8 SGC tissues by Western blot (A) and real-time PCR (B), respectively. *P < 0.05, ** *P *< 0.01, *** *P *< 0.001. **(C) **Expression of AEG-1 protein in each of the primary SGC tissues (T) and adjacent non-cancerous tissues (N) in the same patient determined by Western blot. **(D) **Real time-PCR analysis of AEG-1 expression in each of the T and N tissues. GAPDH was used as an internal control. Columns, mean from three parallel experiments; bars, SD. **(E) **Expression of AEG-1 mRNA in each of the primary SGC tissues (lower panel) and adjacent non-cancerous tissues (upper panel) paired from the same patient determined by IHC.

Western blot analysis revealed that AEG-1 protein was barely detectable in the two normal salivary gland tissues, whereas it was strongly expressed in all eight SGC biopsy tissues (Figure 1A). Real-time PCR was performed to test the mRNA levels of these samples. In consistent with the up-regulated protein levels, all eight SGC tissues exhibited significantly higher levels of AEG-1 mRNA compared with that of the normal salivary gland tissues (Figure 1B).

Further comparative analysis was done in the five pairs of primary SGC tissues and their matched adjacent non-cancerous tissues by Western blot and real-time PCR analysis. The results revealed that the expression level of AEG-1 protein was significantly up-regulated in all five of the SGC tumors (Figure 1C). By real-time PCR analysis, the tumor/adjacent non-cancerous (T/N) ratio of AEG-1 mRNA expression was > 2-fold in all these samples, and the highest ratio was up to about 8-fold (Figure 1D). In the mean time, the expression of the AEG-1 protein was also found to be up-regulated in all 5 human primary SGC tissue samples as compared to the expression in their matched adjacent noncancerous tissues by IHC analyses (Figure 1E).

### Overexpression of AEG-1 protein in archived SGC samples

**Figure 2:**
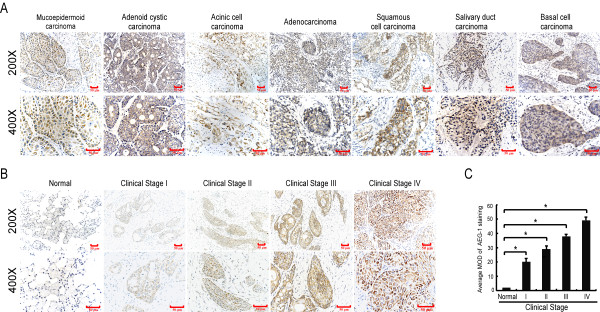
**AEG-1 protein overexpression in archived paraffin-embedded SGC tissue sections as examined by IHC**. **(A) **Representative images from IHC analyses of AEG-1 expression in seven histological types of SGC. **(B) **Representative images from IHC analyses of AEG-1 expression in normal human salivary gland tissues and primary SGC specimens. **(C) **Statistical analyses of the average MOD of AEG-1 staining between normal salivary gland tissues (2 cases) and SGC specimens of different clinical stages. * *P *< 0.05.

To determine the role of AEG-1 in the clinical progression of SGC, IHC analysis was performed in 141 paraffin-embedded, archived SGC tissue samples, including nine histological types of SGC: mucoepidermoid carcinoma, adenoid cystic carcinoma, acinar cell carcinoma, adenocarcinoma, squamous cell carcinoma, salivary duct carcinoma and basal cell carcinoma. AEG-1 protein was positively detected in 96.5% (136/141) of the SGC samples (Table 1) and mainly localized in the cytoplasm of primary cancer cells, which was in consistent with previous reports on AEG-1 expression in other cancer types [10,12,13,25]. As shown in Figure 2A and 2B, the expression of AEG-1 was up-regulated in all the examined histological types of SGC compared with their adjacent normal tissues.

Figure 2B shows representative IHC stained tumor sections of each of the four WHO grades of SGC. Moderate to strong cytoplasmic staining of AEG-1 protein was observed in tumor cells in these primary SGC tissues. By contrast, weak or negative signals were observed in control normal tissues (Figure 2B). Quantitative IHC analysis revealed that the MOD values of AEG-1 staining in all primary SGC were higher than that in control normal tissues and increased along with the progression of tumor grades I to IV (*P *< 0.001, Figure 2C).

### Increased AEG-1 expression correlates with clinicopathologic features of SGC

We further examined the possible correlations between expression levels of AEG-1 and clinical features of SGC. As summarized in Table 2 analyzing of 141 primary SGC samples indicated that AEG-1 expression was strongly correlated with clinical stage (*P *= 0.001), T classification (*P *= 0.008), N classification (*P *= 0.008) and distant metastasis (*P *= 0.006). Spearman correlation analysis (Table 3) proved that high AEG-1 expression level was strongly correlated with advanced clinical stage (R = 0.283, *P *= 0.001), advanced T classification (R = 0.226, *P *= 0.007), lymph node involvement (R = 0.222, *P *= 0.008), and distant metastasis (R = 0.232, *P *= 0.005). However, our analyses did not show significant associations between AEG-1 expression and other clinical features including age, gender, histological type, history of drinking and smoking.

**Table 2:** Correlation between AEG-1 expression and clinicopathologic characteristics of SGC

	Characteristics	AEG-1 expression		Chi-square test *P*-value
		Low or none No. cases (%)	High No. cases (%)	
Gender	Male	42 (29.79)	37 (26.24)	0.160
	Female	20 (14.18)	42 (29.79)	
Age (years)	< 48	32 (22.70)	40 (28.37)	0.908
	≥ 48	30 (21.28)	39 (27.66)	
Clinical Stage	I	10 (7.09)	6 (4.26)	0.001
	II	28 (19.86)	21 (16.31)	
	III	13 (9.22)	21 (14.89)	
	IV	11 (7.80)	31 (21.99)	
T classification	T1	10 (7.09)	7 (4.96)	0.008
	T2	31 (21.99)	29 (20.57)	
	T3	13 (9.22)	20 (14.18)	
	T4	8 (5.67)	23 (16.31)	
N classification	N0	53 (37.59)	53 (37.59)	0.008
	N1	6 (4.26)	11 (7.80)	
	N2	3 (2.13)	15 (10.64)	
M classification	No	51 (36.17)	48 (34.04)	0.006
	Yes	11 (7.8)	31 (21.99)	
Histological Types	Mucoepidermoid carcinoma	13 (9.22)	21 (14.89)	0.48
	Adenoid cystic carcinoma	10 (7.09)	10 (7.09)	
	Acinar cell carcinoma	9 (6.38)	12 (8.51)	
	Adenocarcinoma	8 (5.67)	15 (10.64)	
	Squamous cell carcinoma	6 (4.26)	6 (4.26)	
	Salivary duct carcinoma	10 (7.09)	6 (4.26)	
	Basal cell carcinoma	6 (4.26)	9 (6.38)	
Drinking	No	55(39.01)	62 (43.97)	0.110
	Yes	7(4.96)	17 (12.06)	
Smoking	No	48 (34.04)	53 (37.59)	0.178
	Yes	14 (9.93)	26 (18.44)	

**Table 3:** Spearman correlation analysis between AEG-1 and clinical pathologic factors

Variables	AEG-1 expression level	
	
	Correlation coefficient	*P*-value
Clinical staging	0.283	0.001
T classification	0.226	0.007
N classification	0.222	0.008
M classification	0.233	0.005

### High AEG-1 expression is associated with poor prognosis of patients with SGC

**Figure 3:**
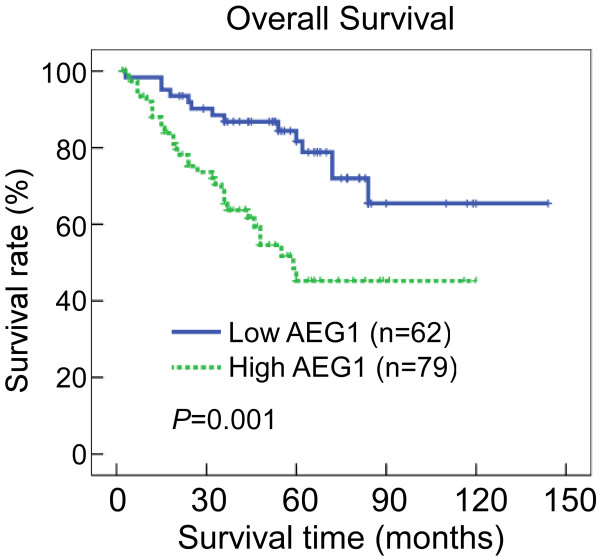
**Kaplan-Meier curves with univariate analyses (log-rank) for patients with low AEG-1 expression (bold line) versus high AEG-1 expression tumors (dotted line)**. The cumulative 5-year survival rate was 78.4% in the low AEG-1 protein expression group (n = 62), whereas it was only 45.0% in the high AEG-1 expression group (n = 79).

Spearman correlation analysis revealed that higher AEG-1 protein levels were associated with shorter survival times (*P *< 0.001), with a correlation coefficient of -0.383. Kaplan-Meier analysis displayed that patients with low AEG-1 expression had longer survival times, whereas those with high AEG-1 expression had shorter survival times (Figure 3, log-rank, *P *= 0.001). The cumulative 5-year survival rate was 78.4% (95% confidence interval, 0.665-0.903) in the low AEG-1 group, compared to only 45.0% (95% confidence interval, 0.303-0.597) in the high AEG-1 group. In addition, multivariate Cox regression analysis revealed that clinical stage, N classification and AEG-1 expression were independent prognostic marker for SGC (Table 4).

**Table 4:** Univariate and multivariate analyses of various prognostic parameters in patients with SGC Cox-regression analysis

	Univariate analysis			Multivariate analysis		
	**No**. patients	*P*	Regression coefficient (SE)	*P*	Relative risk	95% confidence interval
**N classification**		< 0.001	1.319 (0.174)	< 0.001	1.814	1.471-3.283
N0	106					
N1	17					
N2	18					
**Clinical staging**		< 0.001	1.189 (0.197)	< 0.001	2.210	1.454-3.531
I	16					
II	49					
III	34					
IV	42					
**Expression of AEG-1**		0.001	1.047 (0.325)	0.011	2.173	1.231-4.831
Low expression	62					
High expression	79					

**Figure 4:**
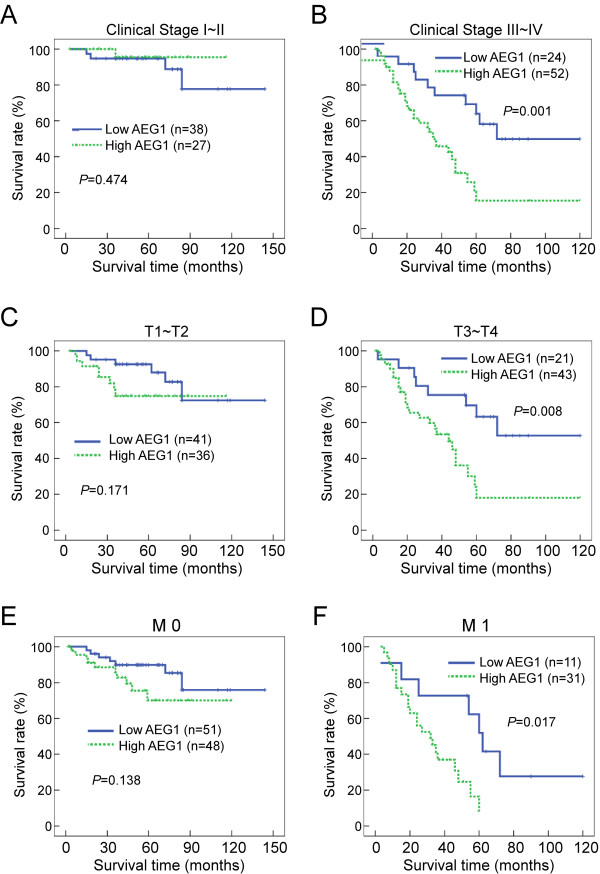
**Overall survival curves stratified by AEG-1 levels according to clinical stage, T and M classifications**. **(A, B) **The differences of survival curves according to AEG-1 expression were seen in advanced clinical stage (B), but not in early clinical stage (A). **(C, D) **In the T3-T4 subgroup, patients with low AEG-1 expression showed significantly better overall survival (D). In the T1-T2 subgroup, the patients' overall survival times were not significantly different between the two groups (C). **(E, F) **Survival time was longer in patients with low AEG-1 expression regardless of distant metastasis (F). No significant difference between low and high AEG-1 expression groups were found in the M0 subgroup (E).

Moreover, the prognostic value of AEG-1 expression was analyzed when stratifying the patients according to the clinical stage, T classification, N classification and M classification. Because only nine samples in subgroups N1-2 exhibited low AEG-1 expression, the overall survival was not analyzed by stratification of N classification. As shown in Figure 4, expression of AEG-1 was strongly associated with overall survival of patients in the late clinical stages. That is, patients with tumors exhibiting high AEG-1 expression had clearly poor survival compared with patients with low AEG-1 expression in the clinical stage III-IV subgroup (Figure 4B, log-rank test, *P *= 0.001). However, in the clinical stage I-II subgroup, no significant difference was found between patients with low and high AEG-1 expression (Figure 4A, log-rank test, *P *= 0.474). Similarly, evidently shorter overall survival time of patients with high AEG-1 expression was revealed in the T3-4 subgroups (Figure 4D, log-rank test, *P *= 0.008), but not in the T1-2 subgroups (Figure 4C, log-rank test, *P *= 0.171). Additionally, shorter overall survival time with high AEG-1 expression was revealed in patients with distant metastasis (Figure 4F, log-rank test, *P *= 0.017), while no such differences was found in patients without distant metastasis (Figure 4E, log-rank test, *P *= 0.138). Thus, AEG-1 seems to be a valuable prognostic marker for patients with late stage or aggressive SGC.

## Discussion

In this study, we presented the first evidence of AEG-1 up-regulation in SGC biopsies at both the mRNA and protein levels compared with adjacent non-cancerous tissues. We also examined 141 SGC that covered a wide spectrum of histological types. AEG-1 protein was observed in 96.5% of archived SGC specimens, and the expression level of AEG-1 protein was found to be significantly correlated with advanced tumor stage and TNM classification, as well as unfavorable prognosis of SGC patients. Our results suggested the important role for AEG-1 protein in the development and progression of SGC.

Due to the rarity and complexity of this type of cancer, it has been difficult to make clinical judgments regarding diagnosis and treatment, as well as the prognosis [1,4,5]. Meanwhile, little is known about its pathogenesis, and few reliable prognostic markers have been identified to predict aggressive biological behavior of SGC. In early studies, HER-2 was found to be an independent marker of poor prognosis of SGC [26,27]. HER-2/neu was found to be amplified and overexpressed in mucoepidermoid carcinomas, as markers of poor prognosis independent of histopathologic grade, tumor size and involvement of regional lymph nodes [27]. The mutated p53 was also shown to be expressed in 7 of 63 (11%) primary SGC in one report [28]. In another study, patients with parotid gland cancer, moderate and high expression of mutated p53 protein were associated more frequently with metastases and poor survival [29]. Other studies also showed that ras-p21 [30] and cyclin D1 [31] are overexpressed in a small subset of SGC, while C-kit is up-regulated in a large percentage of SGC [32,33]. Moreover, vascular endothelial growth factor (VEGF) was found to be up-regulated in 62% of SGC tissues, and its expression is significantly correlated with lymph node metastasis, clinical stage and disease-specific survival [34]. In a previous study, we had found that sphingosine kinase 1 (SPHK1) was associated with SGC progression [35]. In this study, we found that up-regulation of AEG-1 correlated with poor prognosis and reduced survival of patients with SGC. Multivariate analysis showed that AEG-1 protein levels could be used as an independent prognostic predictor for SGC patients, especially in subgroups with advanced clinical stages (III-IV), higher T classification (T3-4), and involvement of distant metastasis (M1). Thus, testing the AEG-1 protein level may be useful for formulating prognosis and guiding the follow-up schedule in SGC patients with advanced and aggressive SGC.

## Conclusions

In this study, we found that up-regulation of AEG-1 correlated with poor prognosis and reduced survival of patients with SGC. Multivariate analysis showed that AEG-1 protein levels could be used as an independent prognostic predictor for SGC patients, especially in subgroups with advanced clinical stages (III-IV), higher T classification (T3-4), and involvement of distant metastasis (M1). Thus, testing the AEG-1 protein level may be useful for formulating prognosis and guiding the follow-up schedule in SGC patients with advanced and aggressive SGC.

## Competing interests

The authors declare that they have no competing interests.

## Authors' contributions

WL carried out Immunohistochemical (IHC) analysis and drafted the manuscript. LG collected the tissue specimens and patient information, and carried out the statistical analyses. YZ carried out the Western blot, RNA extraction and real-time PCR. YW and JL participated in design of the study as well as editing of the manuscript. LS conceived the study, wrote and guided the editing of the manuscript. All authors read and approved the final manuscript.
